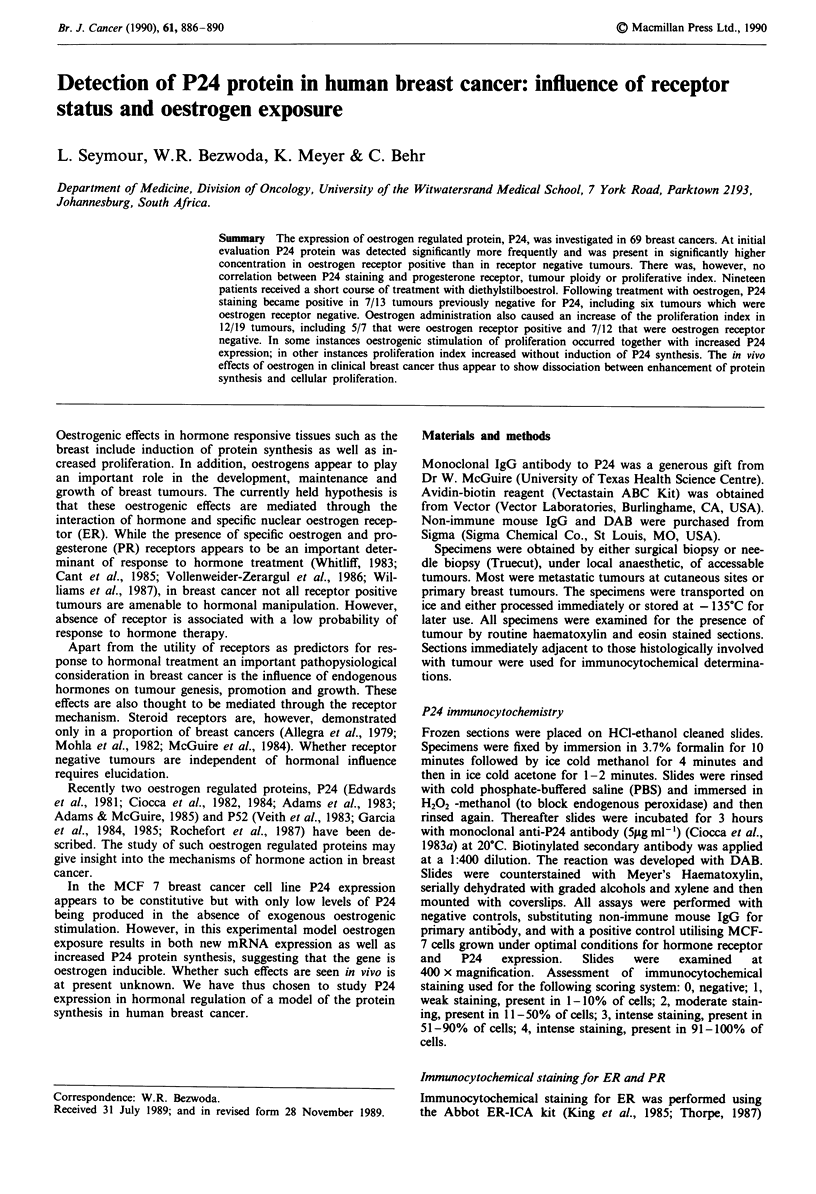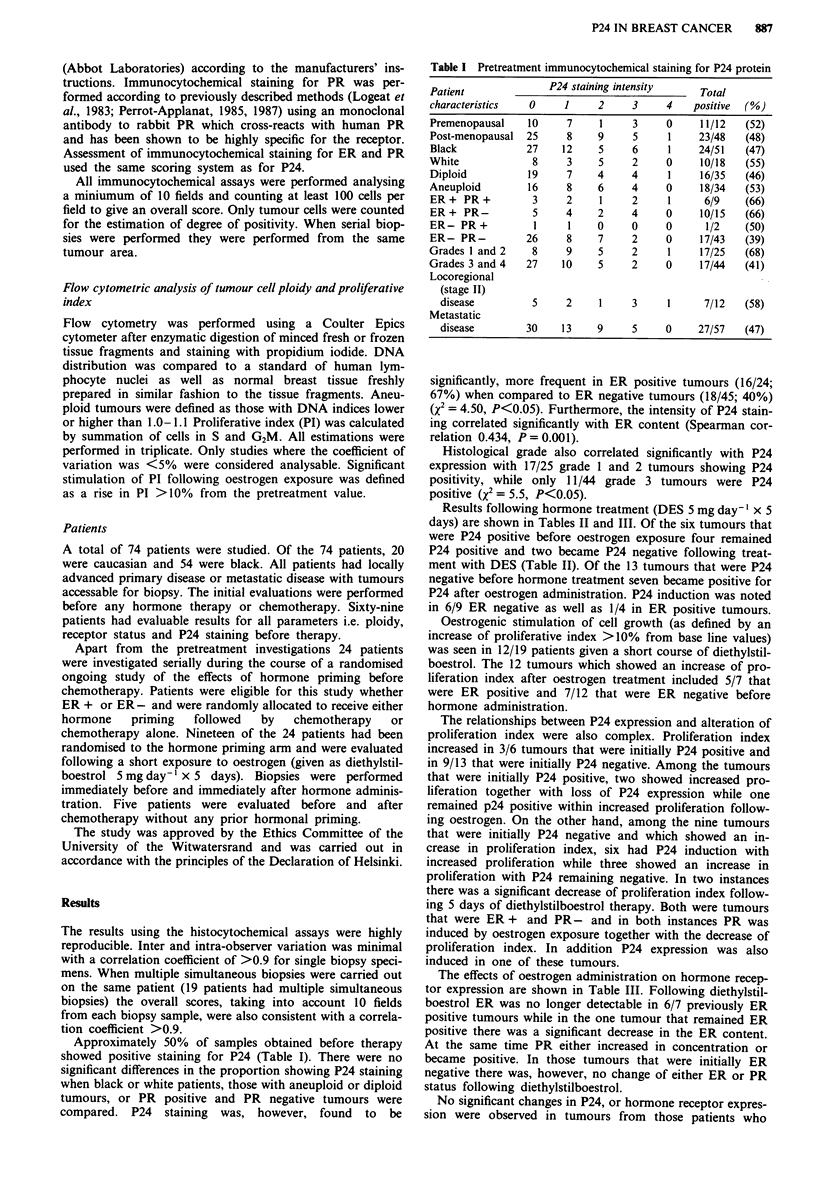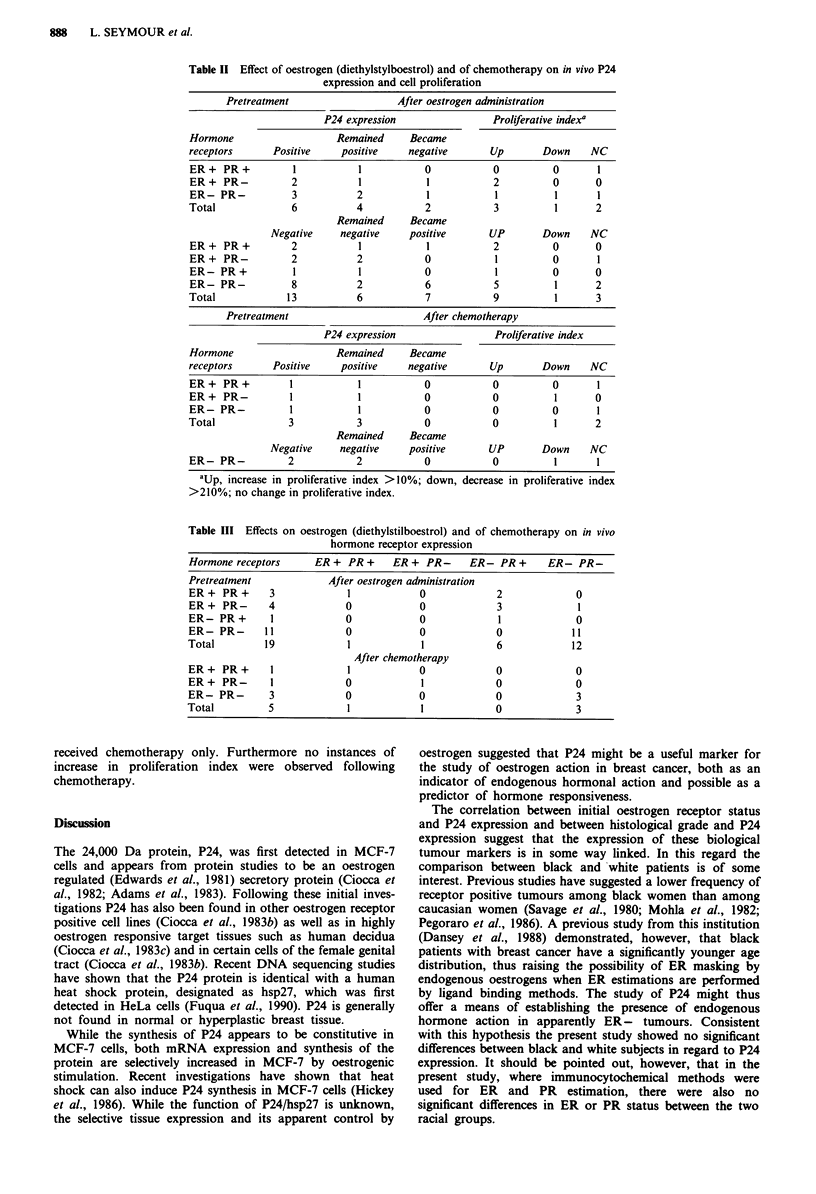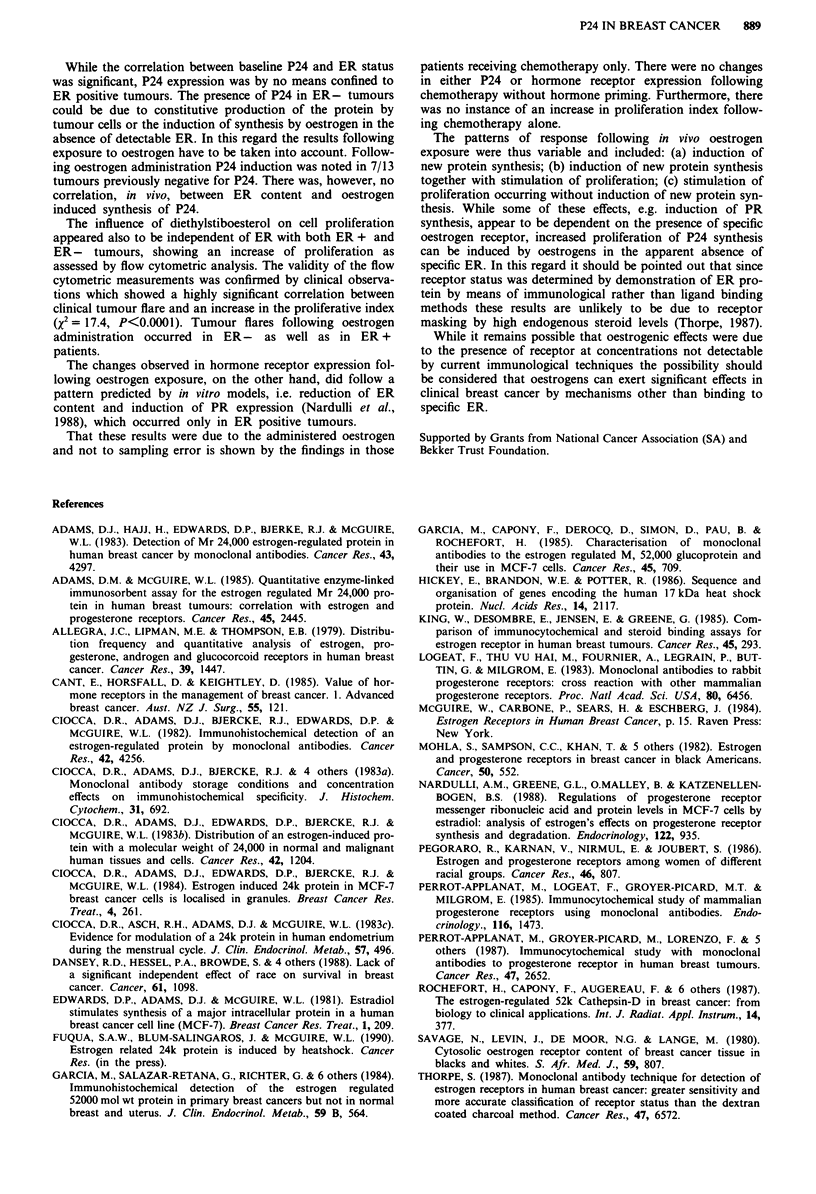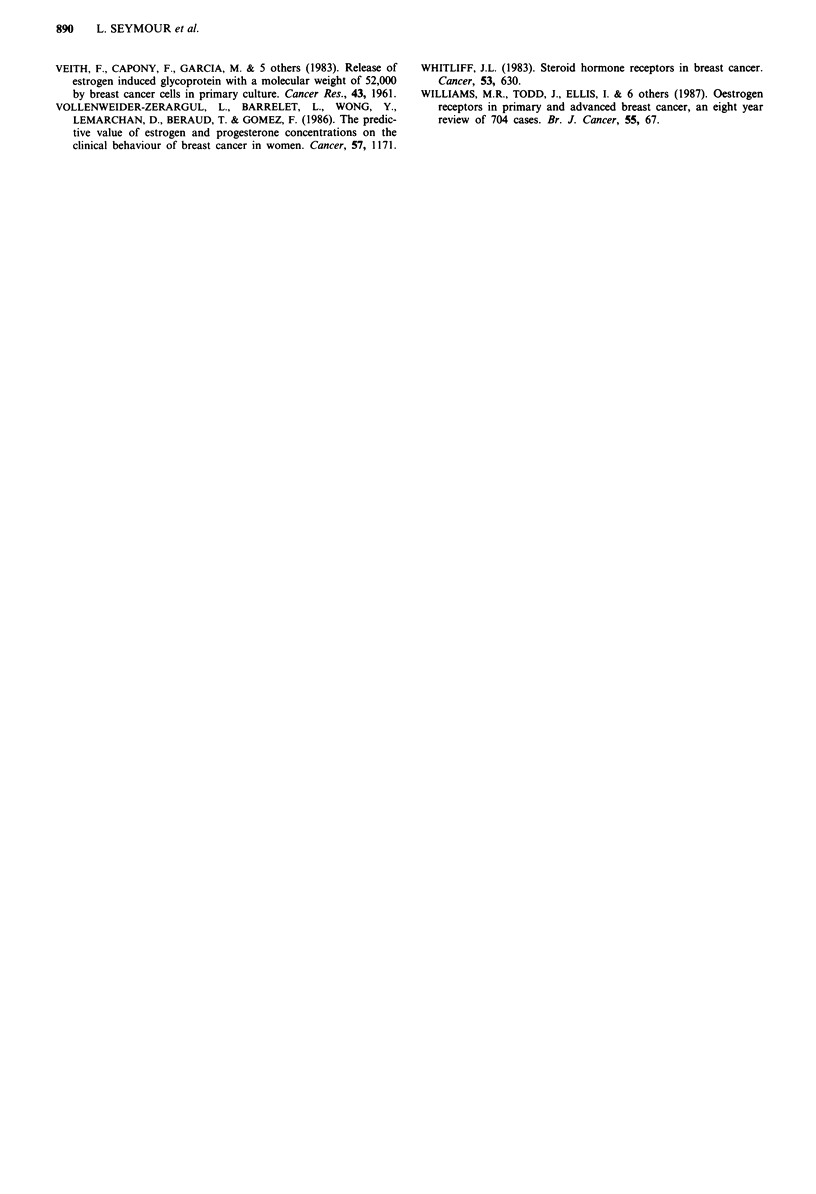# Detection of P24 protein in human breast cancer: influence of receptor status and oestrogen exposure.

**DOI:** 10.1038/bjc.1990.198

**Published:** 1990-06

**Authors:** L. Seymour, W. R. Bezwoda, K. Meyer, C. Behr

**Affiliations:** Department of Medicine, University of the Witwatersrand Medical School, Parktown, Johannesburg, South Africa.

## Abstract

The expression of oestrogen regulated protein, P24, was investigated in 69 breast cancers. At initial evaluation P24 protein was detected significantly more frequently and was present in significantly higher concentration in oestrogen receptor positive than in receptor negative tumours. There was, however, no correlation between P24 staining and progesterone receptor, tumour ploidy or proliferative index. Nineteen patients received a short course of treatment with diethylstilboestrol. Following treatment with oestrogen, P24 staining became positive in 7/13 tumours previously negative for P24, including six tumours which were oestrogen receptor negative. Oestrogen administration also caused an increase of the proliferation index in 12/19 tumours, including 5/7 that were oestrogen receptor positive and 7/12 that were oestrogen receptor negative. In some instances oestrogenic stimulation of proliferation occurred together with increased P24 expression; in other instances proliferation index increased without induction of P24 synthesis. The in vivo effects of oestrogen in clinical breast cancer thus appear to show dissociation between enhancement of protein synthesis and cellular proliferation.


Br. J. Cancer (1990), 61, 886-890

© Macmillan Press Ltd., 1990

### Detection of P24 protein in human breast cancer: influence of receptor status and oestrogen exposure

L. Seymour, W.R. Bezwoda, K. Meyer & C. Behr

*Department of Medicine, Division of Oncology, University of the Witwatersrand Medical School, 7 York Road, Parktown 2193, Johannesburg, South Africa.*

**Summary** The expression of oestrogen regulated protein, P24, was investigated in 69 breast cancers. At initial evaluation P24 protein was detected significantly more frequently and was present in significantly higher concentration in oestrogen receptor positive than in receptor negative tumours. There was, however, no correlation between P24 staining and progesterone receptor, tumour ploidy or proliferative index. Nineteen patients received a short course of treatment with diethylstilboestrol. Following treatment with oestrogen, P24 staining became positive in 7/13 tumours previously negative for P24, including six tumours which were oestrogen receptor negative. Oestrogen administration also caused an increase of the proliferation index in 12/19 tumours, including 5/7 that were oestrogen receptor positive and 7/12 that were oestrogen receptor negative. In some instances oestrogenic stimulation of proliferation occurred together with increased P24 expression; in other instances proliferation index increased without induction of P24 synthesis. The *in vivo* effects of oestrogen in clinical breast cancer thus appear to show dissociation between enhancement of protein synthesis and cellular proliferation.

Oestrogenic effects in hormone responsive tissues such as the breast include induction of protein synthesis as well as increased proliferation. In addition, oestrogens appear to play an important role in the development, maintenance and growth of breast tumours. The currently held hypothesis is that these oestrogenic effects are mediated through the interaction of hormone and specific nuclear oestrogen receptor (ER). While the presence of specific oestrogen and progesterone (PR) receptors appears to be an important determinant of response to hormone treatment (Whitliff, 1983; Cant *et al.*, 1985; Vollenweider-Zerargul *et al.*, 1986; Williams *et al.*, 1987), in breast cancer not all receptor positive tumours are amenable to hormonal manipulation. However, absence of receptor is associated with a low probability of response to hormone therapy.

Apart from the utility of receptors as predictors for response to hormonal treatment an important pathopysiological consideration in breast cancer is the influence of endogenous hormones on tumour genesis, promotion and growth. These effects are also thought to be mediated through the receptor mechanism. Steroid receptors are, however, demonstrated only in a proportion of breast cancers (Allegra *et al.*, 1979; Mohla *et al.*, 1982; McGuire *et al.*, 1984). Whether receptor negative tumours are independent of hormonal influence requires elucidation.

Recently two oestrogen regulated proteins, P24 (Edwards *et al.*, 1981; Ciocca *et al.*, 1982, 1984; Adams *et al.*, 1983; Adams & McGuire, 1985) and P52 (Veith *et al.*, 1983; Garcia *et al.*, 1984, 1985; Rochefort *et al.*, 1987) have been described. The study of such oestrogen regulated proteins may give insight into the mechanisms of hormone action in breast cancer.

In the MCF 7 breast cancer cell line P24 expression appears to be constitutive but with only low levels of P24 being produced in the absence of exogenous oestrogenic stimulation. However, in this experimental model oestrogen exposure results in both new mRNA expression as well as increased P24 protein synthesis, suggesting that the gene is oestrogen inducible. Whether such effects are seen *in vivo* is at present unknown. We have thus chosen to study P24 expression in hormonal regulation of a model of the protein synthesis in human breast cancer.

Correspondence: W.R. Bezwoda.

Received 31 July 1989; and in revised form 28 November 1989.

### Materials and methods

Monoclonal IgG antibody to P24 was a generous gift from Dr W. McGuire (University of Texas Health Science Centre). Avidin-biotin reagent (Vectastain ABC Kit) was obtained from Vector (Vector Laboratories, Burlinghame, CA, USA). Non-immune mouse IgG and DAB were purchased from Sigma (Sigma Chemical Co., St Louis, MO, USA).

Specimens were obtained by either surgical biopsy or needle biopsy (Truecut), under local anaesthetic, of accessable tumours. Most were metastatic tumours at cutaneous sites or primary breast tumours. The specimens were transported on ice and either processed immediately or stored at $-135°C$ for later use. All specimens were examined for the presence of tumour by routine haematoxylin and eosin stained sections. Sections immediately adjacent to those histologically involved with tumour were used for immunocytochemical determinations.

### *P24 immunocytochemistry*

Frozen sections were placed on HCl-ethanol cleaned slides. Specimens were fixed by immersion in 3.7% formalin for 10 minutes followed by ice cold methanol for 4 minutes and then in ice cold acetone for 1-2 minutes. Slides were rinsed with cold phosphate-buffered saline (PBS) and immersed in $H_2O_2$ -methanol (to block endogenous peroxidase) and then rinsed again. Thereafter slides were incubated for 3 hours with monoclonal anti-P24 antibody ($5 \mu g\ ml^{-1}$) (Ciocca *et al.*, 1983*a*) at 20°C. Biotinylated secondary antibody was applied at a 1:400 dilution. The reaction was developed with DAB. Slides were counterstained with Meyer's Haematoxylin, serially dehydrated with graded alcohols and xylene and then mounted with coverslips. All assays were performed with negative controls, substituting non-immune mouse IgG for primary antibody, and with a positive control utilising MCF-7 cells grown under optimal conditions for hormone receptor and P24 expression. Slides were examined at $400 \times$ magnification. Assessment of immunocytochemical staining used for the following scoring system: 0, negative; 1, weak staining, present in 1-10% of cells; 2, moderate staining, present in 11-50% of cells; 3, intense staining, present in 51-90% of cells; 4, intense staining, present in 91-100% of cells.

### *Immunocytochemical staining for ER and PR*

Immunocytochemical staining for ER was performed using the Abbot ER-ICA kit (King *et al.*, 1985; Thorpe, 1987)

(Abbot Laboratories) according to the manufacturers' instructions. Immunocytochemical staining for PR was performed according to previously described methods (Logeat *et al.*, 1983; Perrot-Applanat, 1985, 1987) using an monoclonal antibody to rabbit PR which cross-reacts with human PR and has been shown to be highly specific for the receptor. Assessment of immunocytochemical staining for ER and PR used the same scoring system as for P24.

All immunocytochemical assays were performed analysing a miniumum of 10 fields and counting at least 100 cells per field to give an overall score. Only tumour cells were counted for the estimation of degree of positivity. When serial biopsies were performed they were performed from the same tumour area.

*Flow cytometric analysis of tumour cell ploidy and proliferative index*

Flow cytometry was performed using a Coulter Epics cytometer after enzymatic digestion of minced fresh or frozen tissue fragments and staining with propidium iodide. DNA distribution was compared to a standard of human lymphocyte nuclei as well as normal breast tissue freshly prepared in similar fashion to the tissue fragments. Aneuploid tumours were defined as those with DNA indices lower or higher than 1.0–1.1 Proliferative index (PI) was calculated by summation of cells in S and $G_2M$. All estimations were performed in triplicate. Only studies where the coefficient of variation was <5% were considered analysable. Significant stimulation of PI following oestrogen exposure was defined as a rise in PI >10% from the pretreatment value.

*Patients*

A total of 74 patients were studied. Of the 74 patients, 20 were caucasian and 54 were black. All patients had locally advanced primary disease or metastatic disease with tumours accessable for biopsy. The initial evaluations were performed before any hormone therapy or chemotherapy. Sixty-nine patients had evaluable results for all parameters i.e. ploidy, receptor status and P24 staining before therapy.

Apart from the pretreatment investigations 24 patients were investigated serially during the course of a randomised ongoing study of the effects of hormone priming before chemotherapy. Patients were eligible for this study whether ER + or ER − and were randomly allocated to receive either hormone priming followed by chemotherapy or chemotherapy alone. Nineteen of the 24 patients had been randomised to the hormone priming arm and were evaluated following a short exposure to oestrogen (given as diethylstilboestrol 5 mg day$^{-1}$ × 5 days). Biopsies were performed immediately before and immediately after hormone administration. Five patients were evaluated before and after chemotherapy without any prior hormonal priming.

The study was approved by the Ethics Committee of the University of the Witwatersrand and was carried out in accordance with the principles of the Declaration of Helsinki.

### Results

The results using the histocytochemical assays were highly reproducible. Inter and intra-observer variation was minimal with a correlation coefficient of >0.9 for single biopsy specimens. When multiple simultaneous biopsies were carried out on the same patient (19 patients had multiple simultaneous biopsies) the overall scores, taking into account 10 fields from each biopsy sample, were also consistent with a correlation coefficient >0.9.

Approximately 50% of samples obtained before therapy showed positive staining for P24 (Table I). There were no significant differences in the proportion showing P24 staining when black or white patients, those with aneuploid or diploid tumours, or PR positive and PR negative tumours were compared. P24 staining was, however, found to be significantly, more frequent in ER positive tumours (16/24; 67%) when compared to ER negative tumours (18/45; 40%) ($\chi^2 = 4.50$, $P<0.05$). Furthermore, the intensity of P24 staining correlated significantly with ER content (Spearman correlation 0.434, $P = 0.001$).

**Table I** Pretreatment immunocytochemical staining for P24 protein

| *Patient characteristics* | *P24 staining intensity* | | | | | *Total* | |
|---|---|---|---|---|---|---|---|
| | *0* | *1* | *2* | *3* | *4* | *positive* | *(%)* |
| Premenopausal | 10 | 7 | 1 | 3 | 0 | 11/12 | (52) |
| Post-menopausal | 25 | 8 | 9 | 5 | 1 | 23/48 | (48) |
| Black | 27 | 12 | 5 | 6 | 1 | 24/51 | (47) |
| White | 8 | 3 | 5 | 2 | 0 | 10/18 | (55) |
| Diploid | 19 | 7 | 4 | 4 | 1 | 16/35 | (46) |
| Aneuploid | 16 | 8 | 6 | 4 | 0 | 18/34 | (53) |
| ER + PR + | 3 | 2 | 1 | 2 | 1 | 6/9 | (66) |
| ER + PR − | 5 | 4 | 2 | 4 | 0 | 10/15 | (66) |
| ER − PR + | 1 | 1 | 0 | 0 | 0 | 1/2 | (50) |
| ER − PR − | 26 | 8 | 7 | 2 | 0 | 17/43 | (39) |
| Grades 1 and 2 | 8 | 9 | 5 | 2 | 1 | 17/25 | (68) |
| Grades 3 and 4 | 27 | 10 | 5 | 2 | 0 | 17/44 | (41) |
| Locoregional (stage II) disease | 5 | 2 | 1 | 3 | 1 | 7/12 | (58) |
| Metastatic disease | 30 | 13 | 9 | 5 | 0 | 27/57 | (47) |

Histological grade also correlated significantly with P24 expression with 17/25 grade 1 and 2 tumours showing P24 positivity, while only 11/44 grade 3 tumours were P24 positive ($\chi^2 = 5.5$, $P<0.05$).

Results following hormone treatment (DES 5 mg day$^{-1}$ × 5 days) are shown in Tables II and III. Of the six tumours that were P24 positive before oestrogen exposure four remained P24 positive and two became P24 negative following treatment with DES (Table II). Of the 13 tumours that were P24 negative before hormone treatment seven became positive for P24 after oestrogen administration. P24 induction was noted in 6/9 ER negative as well as 1/4 in ER positive tumours.

Oestrogenic stimulation of cell growth (as defined by an increase of proliferative index >10% from base line values) was seen in 12/19 patients given a short course of diethylstilboestrol. The 12 tumours which showed an increase of proliferation index after oestrogen treatment included 5/7 that were ER positive and 7/12 that were ER negative before hormone administration.

The relationships between P24 expression and alteration of proliferation index were also complex. Proliferation index increased in 3/6 tumours that were initially P24 positive and in 9/13 that were initially P24 negative. Among the tumours that were initially P24 positive, two showed increased proliferation together with loss of P24 expression while one remained p24 positive within increased proliferation following oestrogen. On the other hand, among the nine tumours that were initially P24 negative and which showed an increase in proliferation index, six had P24 induction with increased proliferation while three showed an increase in proliferation with P24 remaining negative. In two instances there was a significant decrease of proliferation index following 5 days of diethylstilboestrol therapy. Both were tumours that were ER + and PR − and in both instances PR was induced by oestrogen exposure together with the decrease of proliferation index. In addition P24 expression was also induced in one of these tumours.

The effects of oestrogen administration on hormone receptor expression are shown in Table III. Following diethylstilboestrol ER was no longer detectable in 6/7 previously ER positive tumours while in the one tumour that remained ER positive there was a significant decrease in the ER content. At the same time PR either increased in concentration or became positive. In those tumours that were initially ER negative there was, however, no change of either ER or PR status following diethylstilboestrol.

No significant changes in P24, or hormone receptor expression were observed in tumours from those patients who

**Table II** Effect of oestrogen (diethylstylboestrol) and of chemotherapy on *in vivo* P24 expression and cell proliferation

| *Pretreatment* | | *After oestrogen administration* | | | | |
|---|---|---|---|---|---|---|
| | | *P24 expression* | | *Proliferative index*[a] | | |
| *Hormone receptors* | *Positive* | *Remained positive* | *Became negative* | *Up* | *Down* | *NC* |
| ER + PR + | 1 | 1 | 0 | 0 | 0 | 1 |
| ER + PR − | 2 | 1 | 1 | 2 | 0 | 0 |
| ER − PR − | 3 | 2 | 1 | 1 | 1 | 1 |
| Total | 6 | 4 | 2 | 3 | 1 | 2 |
| | *Negative* | *Remained negative* | *Became positive* | *UP* | *Down* | *NC* |
| ER + PR + | 2 | 1 | 1 | 2 | 0 | 0 |
| ER + PR − | 2 | 2 | 0 | 1 | 0 | 1 |
| ER − PR + | 1 | 1 | 0 | 1 | 0 | 0 |
| ER − PR − | 8 | 2 | 6 | 5 | 1 | 2 |
| Total | 13 | 6 | 7 | 9 | 1 | 3 |
| *Pretreatment* | | *After chemotherapy* | | | | |
| | | *P24 expression* | | *Proliferative index* | | |
| *Hormone receptors* | *Positive* | *Remained positive* | *Became negative* | *Up* | *Down* | *NC* |
| ER + PR + | 1 | 1 | 0 | 0 | 0 | 1 |
| ER + PR − | 1 | 1 | 0 | 0 | 1 | 0 |
| ER − PR − | 1 | 1 | 0 | 0 | 0 | 1 |
| Total | 3 | 3 | 0 | 0 | 1 | 2 |
| | *Negative* | *Remained negative* | *Became positive* | *UP* | *Down* | *NC* |
| ER − PR − | 2 | 2 | 0 | 0 | 1 | 1 |

[a]Up, increase in proliferative index >10%; down, decrease in proliferative index >210%; no change in proliferative index.

**Table III** Effects on oestrogen (diethylstilboestrol) and of chemotherapy on *in vivo* hormone receptor expression

| *Hormone receptors* | | *ER + PR +* | *ER + PR −* | *ER − PR +* | *ER − PR −* |
|---|---|---|---|---|---|
| *Pretreatment* | | *After oestrogen administration* | | | |
| ER + PR + | 3 | 1 | 0 | 2 | 0 |
| ER + PR − | 4 | 0 | 0 | 3 | 1 |
| ER − PR + | 1 | 0 | 0 | 1 | 0 |
| ER − PR − | 11 | 0 | 0 | 0 | 11 |
| Total | 19 | 1 | 1 | 6 | 12 |
| | | *After chemotherapy* | | | |
| ER + PR + | 1 | 1 | 0 | 0 | 0 |
| ER + PR − | 1 | 0 | 1 | 0 | 0 |
| ER − PR − | 3 | 0 | 0 | 0 | 3 |
| Total | 5 | 1 | 1 | 0 | 3 |

received chemotherapy only. Furthermore no instances of increase in proliferation index were observed following chemotherapy.

### Discussion

The 24,000 Da protein, P24, was first detected in MCF-7 cells and appears from protein studies to be an oestrogen regulated (Edwards *et al.*, 1981) secretory protein (Ciocca *et al.*, 1982; Adams *et al.*, 1983). Following these initial investigations P24 has also been found in other oestrogen receptor positive cell lines (Ciocca *et al.*, 1983*b*) as well as in highly oestrogen responsive target tissues such as human decidua (Ciocca *et al.*, 1983*c*) and in certain cells of the female genital tract (Ciocca *et al.*, 1983*b*). Recent DNA sequencing studies have shown that the P24 protein is identical with a human heat shock protein, designated as hsp27, which was first detected in HeLa cells (Fuqua *et al.*, 1990). P24 is generally not found in normal or hyperplastic breast tissue.

While the synthesis of P24 appears to be constitutive in MCF-7 cells, both mRNA expression and synthesis of the protein are selectively increased in MCF-7 by oestrogenic stimulation. Recent investigations have shown that heat shock can also induce P24 synthesis in MCF-7 cells (Hickey *et al.*, 1986). While the function of P24/hsp27 is unknown, the selective tissue expression and its apparent control by oestrogen suggested that P24 might be a useful marker for the study of oestrogen action in breast cancer, both as an indicator of endogenous hormonal action and possible as a predictor of hormone responsiveness.

The correlation between initial oestrogen receptor status and P24 expression and between histological grade and P24 expression suggest that the expression of these biological tumour markers is in some way linked. In this regard the comparison between black and white patients is of some interest. Previous studies have suggested a lower frequency of receptor positive tumours among black women than among caucasian women (Savage *et al.*, 1980; Mohla *et al.*, 1982; Pegoraro *et al.*, 1986). A previous study from this institution (Dansey *et al.*, 1988) demonstrated, however, that black patients with breast cancer have a significantly younger age distribution, thus raising the possibility of ER masking by endogenous oestrogens when ER estimations are performed by ligand binding methods. The study of P24 might thus offer a means of establishing the presence of endogenous hormone action in apparently ER− tumours. Consistent with this hypothesis the present study showed no significant differences between black and white subjects in regard to P24 expression. It should be pointed out, however, that in the present study, where immunocytochemical methods were used for ER and PR estimation, there were also no significant differences in ER or PR status between the two racial groups.

While the correlation between baseline P24 and ER status was significant, P24 expression was by no means confined to ER positive tumours. The presence of P24 in ER− tumours could be due to constitutive production of the protein by tumour cells or the induction of synthesis by oestrogen in the absence of detectable ER. In this regard the results following exposure to oestrogen have to be taken into account. Following oestrogen administration P24 induction was noted in 7/13 tumours previously negative for P24. There was, however, no correlation, *in vivo*, between ER content and oestrogen induced synthesis of P24.

The influence of diethylstiboesterol on cell proliferation appeared also to be independent of ER with both ER+ and ER− tumours, showing an increase of proliferation as assessed by flow cytometric analysis. The validity of the flow cytometric measurements was confirmed by clinical observations which showed a highly significant correlation between clinical tumour flare and an increase in the proliferative index ($\chi^2 = 17.4$, $P < 0.0001$). Tumour flares following oestrogen administration occurred in ER− as well as in ER+ patients.

The changes observed in hormone receptor expression following oestrogen exposure, on the other hand, did follow a pattern predicted by *in vitro* models, i.e. reduction of ER content and induction of PR expression (Nardulli *et al.*, 1988), which occurred only in ER positive tumours.

That these results were due to the administered oestrogen and not to sampling error is shown by the findings in those patients receiving chemotherapy only. There were no changes in either P24 or hormone receptor expression following chemotherapy without hormone priming. Furthermore, there was no instance of an increase in proliferation index following chemotherapy alone.

The patterns of response following *in vivo* oestrogen exposure were thus variable and included: (a) induction of new protein synthesis; (b) induction of new protein synthesis together with stimulation of proliferation; (c) stimulation of proliferation occurring without induction of new protein synthesis. While some of these effects, e.g. induction of PR synthesis, appear to be dependent on the presence of specific oestrogen receptor, increased proliferation of P24 synthesis can be induced by oestrogens in the apparent absence of specific ER. In this regard it should be pointed out that since receptor status was determined by demonstration of ER protein by means of immunological rather than ligand binding methods these results are unlikely to be due to receptor masking by high endogenous steroid levels (Thorpe, 1987).

While it remains possible that oestrogenic effects were due to the presence of receptor at concentrations not detectable by current immunological techniques the possibility should be considered that oestrogens can exert significant effects in clinical breast cancer by mechanisms other than binding to specific ER.

Supported by Grants from National Cancer Association (SA) and Bekker Trust Foundation.

### References

ADAMS, D.J., HAJJ, H., EDWARDS, D.P., BJERKE, R.J. & MCGUIRE, W.L. (1983). Detection of Mr 24,000 estrogen-regulated protein in human breast cancer by monoclonal antibodies. *Cancer Res.*, **43**, 4297.

ADAMS, D.M. & MCGUIRE, W.L. (1985). Quantitative enzyme-linked immunosorbent assay for the estrogen regulated Mr 24,000 protein in human breast tumours: correlation with estrogen and progesterone receptors. *Cancer Res.*, **45**, 2445.

ALLEGRA, J.C., LIPMAN, M.E. & THOMPSON, E.B. (1979). Distribution frequency and quantitative analysis of estrogen, progesterone, androgen and glucocorcoid receptors in human breast cancer. *Cancer Res.*, **39**, 1447.

CANT, E., HORSFALL, D. & KEIGHTLEY, D. (1985). Value of hormone receptors in the management of breast cancer. 1. Advanced breast cancer. *Aust. NZ J. Surg.*, **55**, 121.

CIOCCA, D.R., ADAMS, D.J., BJERCKE, R.J., EDWARDS, D.P. & MCGUIRE, W.L. (1982). Immunohistochemical detection of an estrogen-regulated protein by monoclonal antibodies. *Cancer Res.*, **42**, 4256.

CIOCCA, D.R., ADAMS, D.J., BJERCKE, R.J. & 4 others (1983*a*). Monoclonal antibody storage conditions and concentration effects on immunohistochemical specificity. *J. Histochem. Cytochem.*, **31**, 692.

CIOCCA, D.R., ADAMS, D.J., EDWARDS, D.P., BJERCKE, R.J. & MCGUIRE, W.L. (1983*b*). Distribution of an estrogen-induced protein with a molecular weight of 24,000 in normal and malignant human tissues and cells. *Cancer Res.*, **42**, 1204.

CIOCCA, D.R., ADAMS, D.J., EDWARDS, D.P., BJERCKE, R.J. & MCGUIRE, W.L. (1984). Estrogen induced 24k protein in MCF-7 breast cancer cells is localised in granules. *Breast Cancer Res. Treat.*, **4**, 261.

CIOCCA, D.R., ASCH, R.H., ADAMS, D.J. & MCGUIRE, W.L. (1983*c*). Evidence for modulation of a 24k protein in human endometrium during the menstrual cycle. *J. Clin. Endocrinol. Metab.*, **57**, 496.

DANSEY, R.D., HESSEL, P.A., BROWDE, S. & 4 others (1988). Lack of a significant independent effect of race on survival in breast cancer. *Cancer*, **61**, 1098.

EDWARDS, D.P., ADAMS, D.J. & MCGUIRE, W.L. (1981). Estradiol stimulates synthesis of a major intracellular protein in a human breast cancer cell line (MCF-7). *Breast Cancer Res. Treat.*, **1**, 209.

FUQUA, S.A.W., BLUM-SALINGAROS, J. & MCGUIRE, W.L. (1990). Estrogen related 24k protein is induced by heatshock. *Cancer Res.* (in the press).

GARCIA, M., SALAZAR-RETANA, G., RICHTER, G. & 6 others (1984). Immunohistochemical detection of the estrogen regulated 52000 mol wt protein in primary breast cancers but not in normal breast and uterus. *J. Clin. Endocrinol. Metab.*, **59 B**, 564.

GARCIA, M., CAPONY, F., DEROCQ, D., SIMON, D., PAU, B. & ROCHEFORT, H. (1985). Characterisation of monoclonal antibodies to the estrogen regulated M, 52,000 glucoprotein and their use in MCF-7 cells. *Cancer Res.*, **45**, 709.

HICKEY, E., BRANDON, W.E. & POTTER, R. (1986). Sequence and organisation of genes encoding the human 17 kDa heat shock protein. *Nucl. Acids Res.*, **14**, 2117.

KING, W., DESOMBRE, E., JENSEN, E. & GREENE, G. (1985). Comparison of immunocytochemical and steroid binding assays for estrogen receptor in human breast tumours. *Cancer Res.*, **45**, 293.

LOGEAT, F., THU VU HAI, M., FOURNIER, A., LEGRAIN, P., BUTTIN, G. & MILGROM, E. (1983). Monoclonal antibodies to rabbit progesterone receptors: cross reaction with other mammalian progesterone receptors. *Proc. Natl Acad. Sci. USA*, **80**, 6456.

MCGUIRE, W., CARBONE, P., SEARS, H. & ESCHBERG, J. (1984). *Estrogen Receptors in Human Breast Cancer*, p. 15. Raven Press: New York.

MOHLA, S., SAMPSON, C.C., KHAN, T. & 5 others (1982). Estrogen and progesterone receptors in breast cancer in black Americans. *Cancer*, **50**, 552.

NARDULLI, A.M., GREENE, G.L., O.MALLEY, B. & KATZENELLENBOGEN, B.S. (1988). Regulations of progesterone receptor messenger ribonucleic acid and protein levels in MCF-7 cells by estradiol: analysis of estrogen's effects on progesterone receptor synthesis and degradation. *Endocrinology*, **122**, 935.

PEGORARO, R., KARNAN, V., NIRMUL, E. & JOUBERT, S. (1986). Estrogen and progesterone receptors among women of different racial groups. *Cancer Res.*, **46**, 807.

PERROT-APPLANAT, M., LOGEAT, F., GROYER-PICARD, M.T. & MILGROM, E. (1985). Immunocytochemical study of mammalian progesterone receptors using monoclonal antibodies. *Endocrinology.*, **116**, 1473.

PERROT-APPLANAT, M., GROYER-PICARD, M., LORENZO, F. & 5 others (1987). Immunocytochemical study with monoclonal antibodies to progesterone receptor in human breast tumours. *Cancer Res.*, **47**, 2652.

ROCHEFORT, H., CAPONY, F., AUGEREAU, F. & 6 others (1987). The estrogen-regulated 52k Cathepsin-D in breast cancer: from biology to clinical applications. *Int. J. Radiat. Appl. Instrum.*, **14**, 377.

SAVAGE, N., LEVIN, J., DE MOOR, N.G. & LANGE, M. (1980). Cytosolic oestrogen receptor content of breast cancer tissue in blacks and whites. *S. Afr. Med. J.*, **59**, 807.

THORPE, S. (1987). Monoclonal antibody technique for detection of estrogen receptors in human breast cancer: greater sensitivity and more accurate classification of receptor status than the dextran coated charcoal method. *Cancer Res.*, **47**, 6572.

VEITH, F., CAPONY, F., GARCIA, M. & 5 others (1983). Release of estrogen induced glycoprotein with a molecular weight of 52,000 by breast cancer cells in primary culture. *Cancer Res.*, **43**, 1961.

VOLLENWEIDER-ZERARGUL, L., BARRELET, L., WONG, Y., LEMARCHAN, D., BERAUD, T. & GOMEZ, F. (1986). The predictive value of estrogen and progesterone concentrations on the clinical behaviour of breast cancer in women. *Cancer*, **57**, 1171.

WHITLIFF, J.L. (1983). Steroid hormone receptors in breast cancer. *Cancer*, **53**, 630.

WILLIAMS, M.R., TODD, J., ELLIS, I. & 6 others (1987). Oestrogen receptors in primary and advanced breast cancer, an eight year review of 704 cases. *Br. J. Cancer*, **55**, 67.